# Land and Seabed Surface Modelling in the Coastal Zone Using UAV/USV-Based Data Integration

**DOI:** 10.3390/s23198020

**Published:** 2023-09-22

**Authors:** Oktawia Specht

**Affiliations:** Department of Transport and Logistics, Gdynia Maritime University, Morska 81-87, 81-225 Gdynia, Poland; oktawiaspecht@gmail.com

**Keywords:** terrain modelling, land surface, seabed surface, geospatial data, coastal zone, Unmanned Aerial Vehicle (UAV), Unmanned Surface Vehicle (USV)

## Abstract

The coastal zone is an area that includes the sea coast and adjacent parts of the land and sea, where the mutual interaction of these environments is clearly marked. Hence, the modelling of the land and seabed parts of the coastal zone is crucial and necessary in order to determine the dynamic changes taking place in this area. The accurate determination of the terrain in the coastal zone is now possible thanks to the use of Unmanned Aerial Vehicles (UAVs) and Unmanned Surface Vehicles (USVs). The aim of this article is to present land and seabed surface modelling in the coastal zone using UAV/USV-based data integration. Bathymetric and photogrammetric measurements were carried out on the waterbody adjacent to a public beach in Gdynia (Poland) in 2022 using the DJI Phantom 4 Real Time Kinematic (RTK) UAV and the AutoDron USV. As a result of geospatial data integration, topo-bathymetric models in the coastal zone were developed using the following terrain-modelling methods: Inverse Distance to a Power (IDP), kriging, Modified Shepard’s Method (MSM) and Natural Neighbour Interpolation (NNI). Then, the accuracies of the selected models obtained using the different interpolation methods, taking into account the division into land and seabed parts, were analysed. Research has shown that the most accurate method for modelling both the land and seabed surfaces of the coastal zone is the kriging (linear model) method. The differences between the interpolated and measurement values of the R95 measurement are 0.032 m for the land part and 0.034 m for the seabed part. It should also be noted that the data interpolated by the kriging (linear model) method showed a very good fit to the measurement data recorded by the UAVs and USVs.

## 1. Introduction

The coastal zone is an area that includes the sea coast and adjacent parts of the land and sea, where the mutual interaction of these environments is clearly marked [1,2,3]. Based on data provided by the General Bathymetric Chart of the Oceans (GEBCO), it appears that the coastal zone is one of the most dynamically changing regions on Earth [4], in particular, in the coastal zone [5,6]. Changes in coastal zone topography are caused by anthropogenic and natural factors, among which, the following can be distinguished: biological activity, marine erosion, rising water level [7], ocean currents, rock debris transport, tides, wave action [8], seawater intrusion [9], earthquakes, river regulation [10], ocean acidification, rising temperatures [11] and coastal flooding [12]. Seabed changes taking place in the coastal zone result in the need to carry out topo-bathymetric monitoring in these areas in order to prevent negative effects on the water environment and humans [13,14,15].

The topo-bathymetric monitoring of the coastal zone is possible thanks to the use of hydroacoustic and optoelectronic devices and systems [16]. The operation of hydroacoustic systems involves sending a high-frequency sound wave into the water environment and registering the vibration of the wave reflected by the object. Most hydroacoustic measurement systems are equipped with the following devices: echo sounders, hydrometric stations, Inertial Navigation Systems (INSs), satellite navigation systems, sonar and Sound Velocity Profilers (SVPs) [17,18,19,20,21,22]. However, the operation of optoelectronic systems involves the use of specific properties of light in order to collect, obtain, present, process and transmit information, including geospatial information. Most optoelectronic measurement systems are equipped with image sensors, INSs, laser rangefinders, Light Detection and Ranging (LiDAR) systems, Radio Detection and Ranging (RADAR) systems and satellite navigation systems [23,24,25,26,27,28].

Ibrahim et al. [29] developed a topo-bathymetric surface model using a Triangulation Irregular Network (TIN). The aim of their article was to determine the volume of a reservoir that was subject to sedimentation and siltation. The research was carried out on the Tunga Dam in Nigeria, which is located at a small hydroelectric power plant with a generating capacity of approximately 400 kW. The Hi-Target V30 Differential Global Positioning System (DGPS) receiver and the EchoMap 50 s Single Beam Echo Sounder (SBES) were used during the realisation of the bathymetric and topographic measurements. Next, a Digital Elevation Model (DEM) was modelled using a TIN algorithm. Before the dam capacity calculation was started, it was decided to determine the accuracy of the surface interpolation. For this purpose, the cross-validation of measured and estimated points was applied. The accuracy of the topo-bathymetric surface interpolation was as follows: 0.102 m (Mean Error (ME)), 0.126 m (Mean Square Error (MSE)) and 0.354 m (Root Mean Square Error (RMSE)). The research showed that the deepest point of the dam was 21.25 m, and the volumetric capacity of the reservoir amounted to 19,339,627.64 m^3^. Moreover, the Digital Depth Model (DDM) indicates the meandering nature of the dam, which makes it necessary to perform dredging in order to enhance the reservoir’s capacity.

Lubczonek et al. [30] proposed a method of integrating data obtained using an Unmanned Aerial Vehicle (UAV) and an Unmanned Surface Vehicle (USV). The aim of the article was to develop a bathymetric chart taking into account depths up to the shoreline. The research used the DJI Phantom 4 Pro UAV equipped with a Complementary Metal–Oxide–Semiconductor (CMOS) camera with a resolution of 20 Mpx and a Global Navigation Satellite System (GNSS) receiver (positioning accuracy of 0.5–1.5 m), as well as the Gerris USV, on which the Echologger EU400 SBES, the GNSS Real Time Kinematic (RTK) EMLID Reach M2 receiver and an Inertial Measurement Unit (IMU) were mounted. Moreover, Ground Control Points (GCPs), determined using the GNSS RTK geodetic method, were used for the georeferencing of images taken by the drone. The study was carried out on Lake Dąbie (Poland), with an average depth of 2.61 m. Data obtained using UAVs and USVs were subjected to a harmonisation process in order to create a Digital Bathymetric Model (DBM) of the waterbody. Five methods were used to model the seabed topography: kriging, Natural Neighbour Interpolation (NNI), Inverse Distance to a Power (IDP), radial basis function and triangulation. The research showed that the accuracy of land surface modelling using the above-mentioned methods is high (ME = 0.01 m, RMSE = 0.03 m). Therefore, it can be concluded that the data obtained using unmanned measurement platforms can be used to create navigation charts of shallow (coastal) waters; the analysis of seabed topography at hydrotechnical structures; and archaeological mapping.

Wang et al. [31] used an Airborne LiDAR Bathymetry (ALB) mounted on a UAV to determine the depth of shallow waterbodies and detect objects. The aim of the article was to present and evaluate the possibility of using an ALB system in hydrographic surveys. As part of the research, a lightweight dual-wavelength topo-bathymetric LiDAR system (Mapper4000U) generating near-infrared and green pulses at a frequency of 4 kHz was used, which was mounted on a DJI Matrice 600 Pro UAV. Bathymetric measurements were carried out on Dazhou Island (China), whose water is clear, and its Secchi Depth (SD) is in a range of 5–10 m. Validation studies were conducted based on the MultiBeam EchoSounder (MBES) (Hydro-tech Marine MS400) registrations. The research showed that lightweight UAV-borne topo-bathymetric LiDAR is suitable for determining a depth of 1.7–1.9 Secchi depths. Moreover, the accuracy of the water bottom was 0.1268 m (RMSE) and 0.3 m (*p* = 0.98), and the fitting precision of the water surface amounted to 0.1227 m (RMSE). The ALB system showed high-spatial-resolution geospatial data with an average point density of 42 points/m^2^. The study also proved that, on the basis of the seabed point cloud, the existence of a 1 m target cube and the rough shape of a 2 m target cube are easily detected at a depth of 12 m.

Based on a review of the literature, it appears that modelling the land and seabed parts of the coastal zone is crucial and necessary in order to determine the dynamic changes taking place in this area. Therefore, the main aim of this article is to present the accuracy obtained using a topo-bathymetric model separately for land and seabed surfaces in the coastal zone.

This article has the following structure. Section 2 describes the measurement location, as well as the realisation of bathymetric and topographic measurements using UAVs and USVs. Moreover, this section presents how geospatial data recorded by unmanned measurement platforms were elaborated. Section 3 shows how topo-bathymetric models in the coastal zone were developed using the following terrain modelling methods: IDP, kriging, Modified Shepard’s Method (MSM) and NNI. Then, the accuracies of selected models obtained by different interpolation methods, taking into account the division into land and seabed parts, were analysed. The paper concludes with final (general and detailed) conclusions that summarise its contents.

## 2. Materials and Methods

### 2.1. Measurement Place

**Figure 1:**
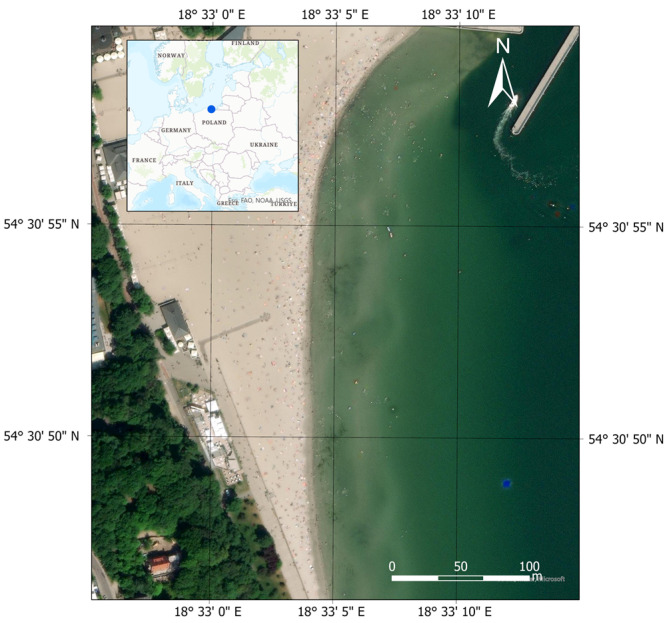
The location of topo-bathymetric measurements conducted on 10 June 2022 on the waterbody at the public beach in Gdynia.

Topo-bathymetric measurements were carried out on the waterbody at a public beach in Gdynia (Figure 1). It is located in proximity to the Mariusz Zaruski Marina in Gdynia. This waterbody has a typical running coastline (a 400-metre-straight sandy section), and the depths increase with distance from the shore. However, according to previously conducted research [26], in certain locations, there are alternating “shallows” and “depressions” that appear up to 1 m isobath. The above-mentioned seafloor relief changes are the result of the activities of the Maritime Office in Gdynia, which refills the waterbody with material (sand) acquired from the dredging of port approach fairways. The visibility depth of the Secchi disk, analysed in the years 2014–2015 in the waters of the Port of Gdynia, was approximately 2 m [32,33].

### 2.2. Photogrammetric Data

The acquisition of data from photogrammetric images is a method commonly used in the coastal zone, which enables the registration of data from areas that cannot be accessed by geodetic and hydrographic devices [34]. Therefore, for this study, a section of the public beach in Gdynia, along with the waterbody adjacent to it, was surveyed using DJI Phantom 4 RTK UAV. Photogrammetric data preparation was initiated with the import of the drone images taken. The photos were recorded in the World Geodetic System 1984 (WGS 84)/Universal Transverse Mercator (UTM) system, with the UTM zone being calculated based on Exchangeable Image File Forma (EXIF) data. Subsequently, the georeference point coordinates, which were originally recorded in the PL-2000 plane coordinate system and the PL-EVRF2007-NH height system, were transformed. As for the bathymetric data, the target horizontal datum was the PL-UTM system, while the heights remained in the PL-EVRF2007-NH system. It is worth noting that the images acquired from the UAV originally had georeferences. However, it is recommended that georeferencing be performed based on the georeference point coordinates for each photogrammetric measurement.

**Figure 2:**
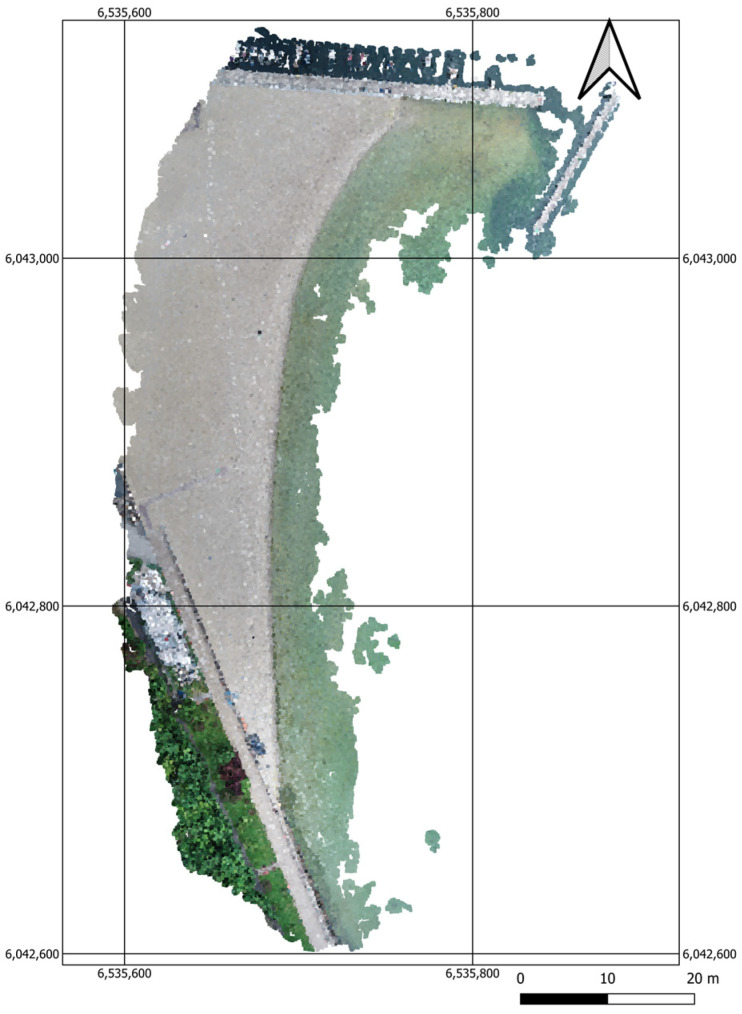
A map showing a point cloud for the waterbody adjacent to the public beach in Gdynia.

The next stage of work involved the generation of a point cloud using the Pix4Dmapper software (Figure 2).

This was followed by the main process of georeferencing. In the Pix4Dmapper software, the process involves assigning the coordinates determined by the GNSS RTK receiver to the images in the point cloud, in which the georeference points are visible. Then, the transformation of coordinates for the entire point cloud is performed.

Based on Table 1, it can be noted that the average shift of the easting coordinate in the PL-2000 and PL-UTM systems was 0.012 m. However, shifts of 0.006 m and 0.013 m were recorded for the northing and normal-height coordinates, respectively. As can be seen, the data recorded by the UAV has a high degree of accuracy.

For the transformation of UAV point cloud coordinates, the Pix4Dmapper software uses a seven parameter transformation. This is a conformal transformation, which preserves the shape of the data. It transforms a set of points into another by changing its rotation, scaling and translation. The mathematical model of the seven-parameter transformation is founded on the transformation of coordinates based on previously determined parameters such as the scale factor, rotation matrices and translation vectors. These parameters are calculated using the relationships between the points recorded in two systems: the primary (point cloud) and the secondary (georeference points).

**Figure 3:**
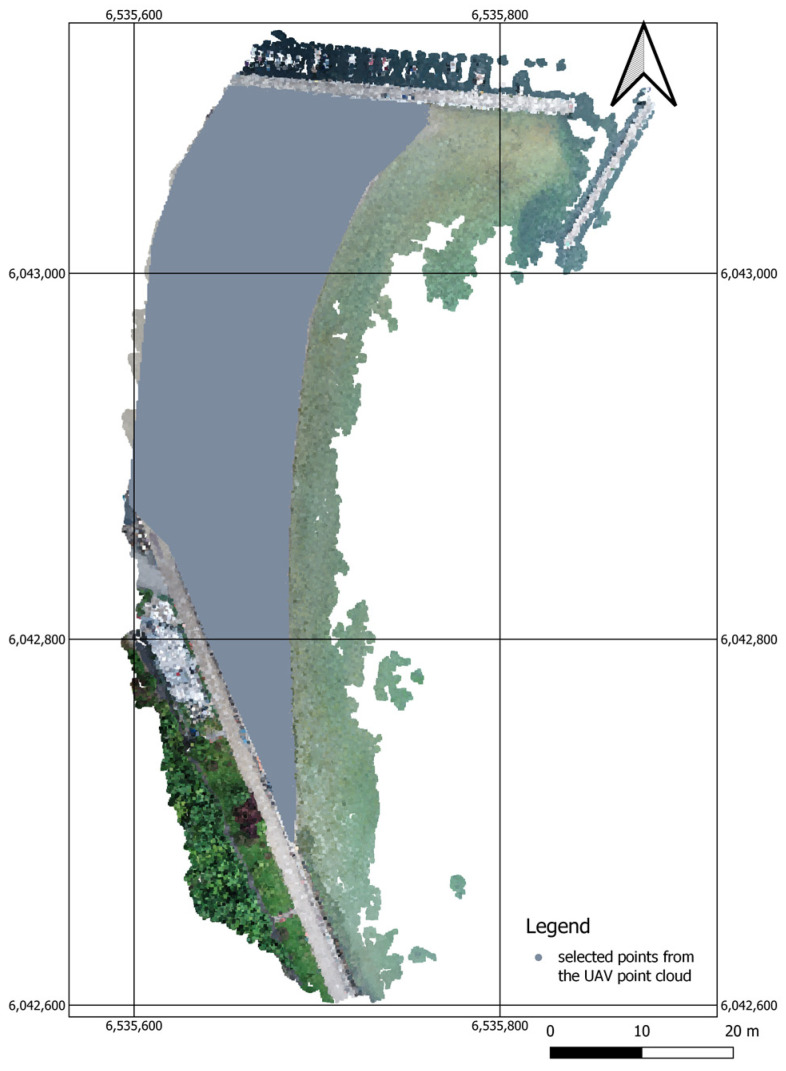
A map showing the points extracted from the UAV point cloud in an orthophotomap.

The UAV point cloud, which had a correct coordinate system, was then converted into an a.xyz file. The UAV point cloud was generated in the form of a GRID DEM Surface Model with a grid spacing of 1 m and was subsequently reconstructed using the CloudCompare software to cut only the beach (grey colour) from it (Figure 3).

### 2.3. Bathymetric Data

**Figure 4:**
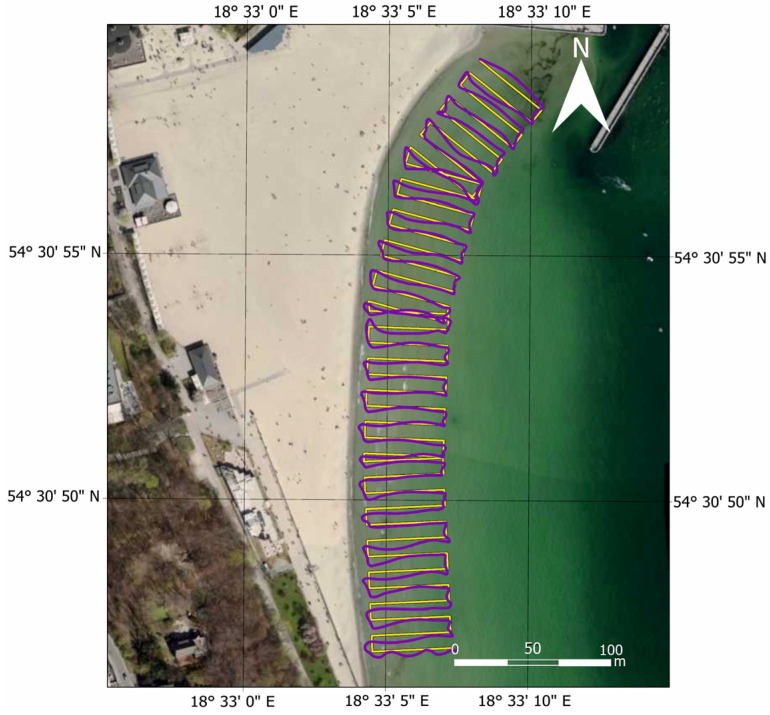
A map showing the distribution of the bathymetric data covering the waterbody adjacent to the public beach in Gdynia after the preliminary data-cleaning process. Own study based on [35].

Following the bathymetric measurements covering the waterbody adjacent to the public beach in Gdynia, performed using the AutoDron USV, it was necessary to analyse the bathymetric data (Figure 4).

The bathymetric data were assigned coordinates from differential GNSS RTK measurements in the PL-2000 system, while the depths recorded by the echo sounder were obtained in the PL-EVRF2007-NH system. It was decided that the target horizontal datum for the bathymetric data would be the PL-UTM plane coordinate system, while the heights would be expressed in the PL-EVRF2007-NH normal height system. Therefore, before data elaboration, the coordinates were transformed from the PL-2000 system to the PL-UTM system. The transformation was carried out using the QGiS software.

The bathymetric data were assigned coordinates from differential GNSS RTK measurements in the PL-2000 system, while the depths recorded by the echo sounder were obtained in the PL-EVRF2007-NH system. It was decided that the target horizontal datum for the bathymetric data would be the PL-UTM plane coordinate system, while the heights would be expressed in the PL-EVRF2007-NH normal height system. Therefore, before data elaboration, the coordinates were transformed from the PL-2000 system to the PL-UTM system. The transformation was carried out using the QGiS software.

Depths recorded erroneously by an SBES were then deleted. Depths can have erroneous values because, in shallow depths (less than 30 cm), the phenomenon of multiple reflections of a hydroacoustic signal often occurs. The recorded depths are then greater than they actually are. The point cloud cleaning was performed using the CloudCompare and QGiS software.

**Figure 5:**
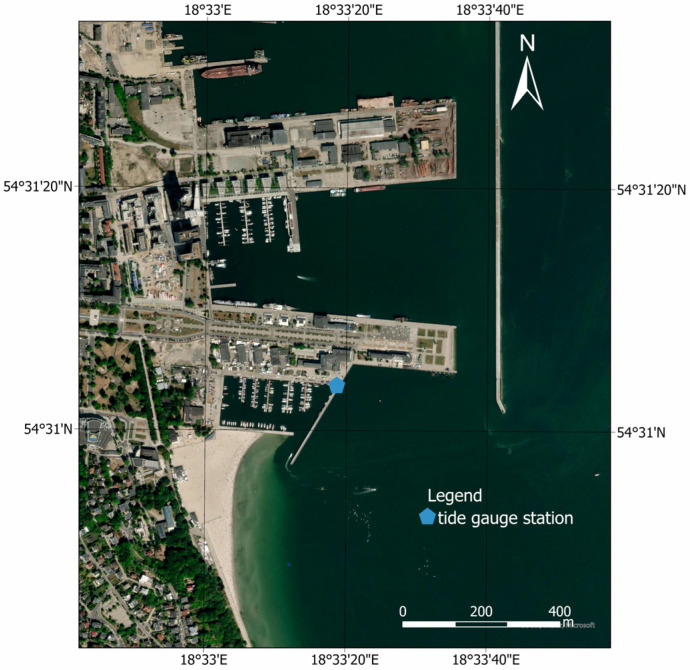
The location of the tide gauge station in Gdynia.

Subsequently, the depths were referred to the so-called chart datum, which, for the PL-EVRF2007-NH system for a tide gauge station located in Gdynia (Figure 5), amounts to 491.3 cm. It should be noted that a separate zero ordinate applies to all tide gauge stations along the Polish coast.

The formula for the normal height of a point measured by an echo sounder in the PL-EVRF2007-NH height system is as follows [36]:

(1) $$d’=-\left(d+\Delta d_{ET}\pm \Delta d_{CD}\right)$$

where

*d’*—normal height of the point measured by the echo sounder in the PL-EVRF2007-NH height system (cm);
*d*—depth measured by the echo sounder (cm);
Δ*d_ET_*—draft of the echo sounder transducer (cm);
Δ*d_CD_*—depth correction referring to the chart datum in the PL-EVRF2007-NH height system (cm), which must added be if the averaged water level (${\bar{d}}_{SL}$) is less than 491.3 cm. Otherwise, the depth correction must be subtracted.

The depth correction *Δd_CD_* is defined as follows [36]:

(2) $$\Delta d_{CD}=491.3\;cm-{\bar{d}}_{SL}$$

where

${\bar{d}}_{SL}$—averaged sea level observed on the mareograph between consecutive full hours in the PL-EVRF2007-NH height system (cm).

The above stage is crucial in processing bathymetric data from marine areas where water levels impact depth measurements. In regions with tidal activity, it is essential to account for this factor in data elaboration. The resulting bathymetric data had values ranging from –1.37 m to –0.21 m.

### 2.4. Topo-Bathymetric Data Integration Models

After importing the bathymetric and photogrammetric data to the PL-UTM (zone 34N) and PL-EVRF2007-NH systems, it was possible to generate Digital Terrain Models (DTMs) of the coastal zone. For this purpose, the Surfer software was used, which enables the representation of a surface model in the form of a regular square grid (GRID) [37]. It is a form of numerical representation of a terrain model in the form of a grid of squares covering the area evenly. GRID models are created as a result of interpolation, i.e., the process of estimating an unknown value between known values using specific interpolation methods. Then, GRID nodes are generated, which form a structure of regular rectangles (usually squares) with a fixed resolution [2]. In total, 28,990 points of the land surface recorded by the UAV and 5136 points of the seabed terrain recorded by the USV were used to create a grid of squares. For all analysed models, the length of the GRID side was assumed to be 1 m for the northing coordinate and 1 m for the easting coordinate. As a result, a grid of squares consisting of 412 rows and 213 columns was obtained, resulting in a total of 87,756 GRID nodes. It should be noted that not all methods managed to obtain elevation/depth data in each node of the grid of squares.

To generate topo-bathymetric models of the coastal zone, we decided to use the most commonly used terrain interpolation methods, among which, the following can be distinguished [2,38]:

1. IDW is the simplest deterministic method, the basis of which is the direct statement that geographic objects located closer to each other are more similar than those located further away. The value at a given location is determined based on nearby points with known values, which are weighted by a factor proportional to the inverse of their distance [39,40];
2. The IDP method, which involves calculating the weighted average of observations in the surroundings. The observation weights are inversely proportional to the distance between the measurement points and the interpolated point [41,42,43];
3. MSM, which is a generalisation of the inverse distance method. The algorithm uses two types of interpolation functions: faithful, in which the function parameter is consistent with the measured parameter, and smoothing, in which the input value is not precisely located on the generated surface [44,45,46];
4. The kriging method is an interpolation method based on geostatistics, in which an interpolation error called a kriging variance is determined. The kriging algorithm is effective because it can compensate for the data in the set by giving those areas less weight in the overall prediction. It also allows for extrapolation beyond the data area [47,48];
5. NNI is a method based on the Voronoi tessellation of a discrete set of spatial points. This has advantages over simpler interpolation methods, such as nearest-neighbour interpolation, in that it provides a smoother approximation of the underlying true function [49,50].

Following the generation of the GRID network, the statistical parameters for individual DTMs of the GRID type were calculated: Root Mean Square (RMS) (m), min. and max interpolated value (m), range (R) (m) and InterQuartile Range (IQR) (m). The IQR is calculated as follows:

(3) $$IQR=Q_{3}\left(\hat{z}\right)-Q_{1}\left(\hat{z}\right)$$

where

$Q_{1}\left(\hat{z}\right)$—the first quartile (25th empirical quartile) of the $\hat{z}$ value (m);
$Q_{3}\left(\hat{z}\right)$—the third quartile (75th empirical quartile) of the $\hat{z}$ value (m).

Subsequently, it was possible to assess the accuracy of the land and seabed surfaces of the coastal zone. As the criteria for assessing the accuracy of the modelled surfaces, the difference between the height coordinates measured by the UAV or USV and the modelled height coordinates for the same plane coordinates was adopted. In turn, on this basis, the following measures of terrain modelling methods were calculated [51,52,53]:

(4) $$RMSE=\sqrt{\frac{\sum_{i}^{n}{\left({\hat{z}}_{i}-z_{i}\right)}^{2}}{n}}$$

(5) $$MAE=\frac{\sum_{i}^{n}\left|{\hat{z}}_{i}-z_{i}\right|}{n}$$

(6) $$R^{2}=\frac{\sum_{i}^{n}{\left({\hat{z}}_{i}-\bar{z}\right)}^{2}}{\sum_{i}^{n}{\left(z_{i}-\bar{z}\right)}^{2}}$$

where

RMSE—Root Mean Square Error (m);
*n* –number of measurement points (–);
*i*—number representing successive measurement points (–);
*z_i_*—height of the *i*-th point measured by the UAV or USV (m);
${\hat{z}}_{i}$—interpolated value of *z_i_* (m);
MAE—Mean Absolute Error (m);
R^2^—coefficient of determination (–);
$\bar{z}$—arithmetic mean of *z*-value (m).

In addition, two accuracy measures (R68 and R95) were additionally calculated, which are determined on the basis of sorting the study variable from smallest to largest. The computed height differences were sorted (from smallest to largest) and based on them; an error value that is greater than exactly 68% (1*σ*) and 95% (2*σ*) of the height difference population was determined. The advantage of the R68 and R95 accuracy measures is that the analysed variable is not normally distributed [54,55].

## 3. Results

### 3.1. Modelling the Land Surface of the Coastal Zone

#### 3.1.1. IDP (*p* = 1)

**Figure 6:**
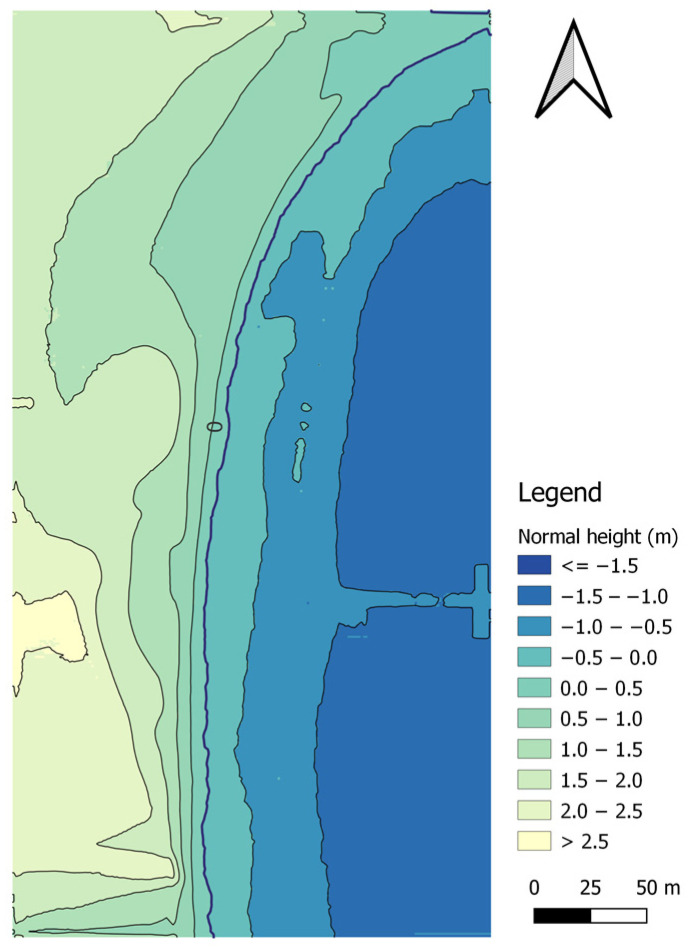
A topo-bathymetric chart of the waterbody adjacent to the public beach in Gdynia, obtained using the IDP (*p* = 1) method.

The first method of land surface modelling was the IDP method, with an exponent of *p* = 1. The topo-bathymetric model for the IDP method (*p* = 1) was created based on 34,166 measurement points and had 87,756 nodes, of which 44,254 nodes covered the land surface of the coastal zone. In total, 29,030 measurement points were used to model the topography of the land surface, which were compared with the corresponding points (with the same flat coordinates) from the interpolated DTM using the IDP (*p* = 1) method (Figure 6).

The RMS value of heights interpolated by the IDP (*p* = 1) method was 1.527 m. The min. height value amounted to 0.000 m, while the max value was 2.690 m. The range amounted to 2.690 m, while the IRQ was 0.828 m.

The accuracy of the interpolated DTM in relation to the measurements was determined using the RMSE to be 0.061 m and the MAE to be 0.023 m. The RMSE and MAE values indicate a small difference between the interpolated and measurement values. The coefficient of determination was obtained at a level of 0.992, which means that the fit of the model to the measurement data is very good. The difference between the interpolated and measurement values for the R68 measure is 0.019 m, while for the R95 measure, it has a value of 0.072 m. It can be assumed that the application of a modified IDW algorithm in the form of the IDP (*p* = 1) method only for land surfaces can yield better accuracies for the model.

#### 3.1.2. IDP (*p* = 2)

**Figure 7:**
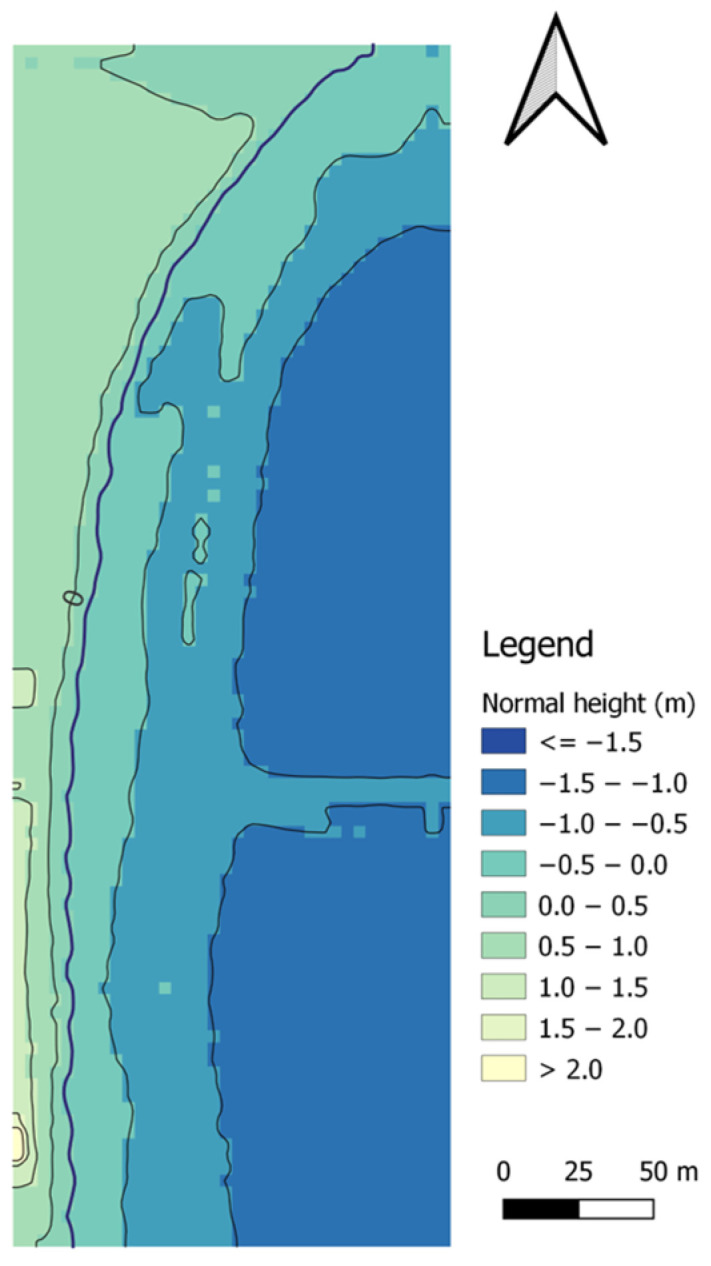
A topo-bathymetric chart of the waterbody adjacent to the public beach in Gdynia, obtained using the IDP (*p* = 2) method.

Subsequently, we decided to use the IDP method again to model the land surface of the coastal zone, only with an increased exponent of *p* = 2. The topo-bathymetric model for the IDP method (*p* = 2) was created based on 34,166 measurement points and had 87,756 nodes, of which 44,464 nodes covered the land surface of the coastal zone. In total, 29,030 measurement points were used to model the topography of the land surface, which were compared with the corresponding points (with the same flat coordinates) from the interpolated DTM using the IDP (*p* = 2) method (Figure 7).

The RMS value of heights interpolated by using the IDP (*p* = 2) method was 1.522 m. The min. height value amounted to 0.000 m, while the max value was 3.102 m. The range amounted to 3.102 m, while the IRQ was 0.851 m.

The accuracy of the interpolated DTM in relation to the measurements was determined using the RMSE to be 0.042 m and the MAE to be 0.016 m. The RMSE and MAE values indicate a small difference between the interpolated and measurement values. The coefficient of determination was obtained at a level of 0.996, which means that the fit of the model to the measurement data is very good. The difference between the interpolated and measurement values for the R68 measure is 0.013 m, while for the R95 measure, it has a value of 0.048 m. Based on the statistical analysis, it can be concluded that, with the growth of the exponent in the IDP method, the accuracy of modelling the land surface of the coastal zone increases.

#### 3.1.3. MSM

**Figure 8:**
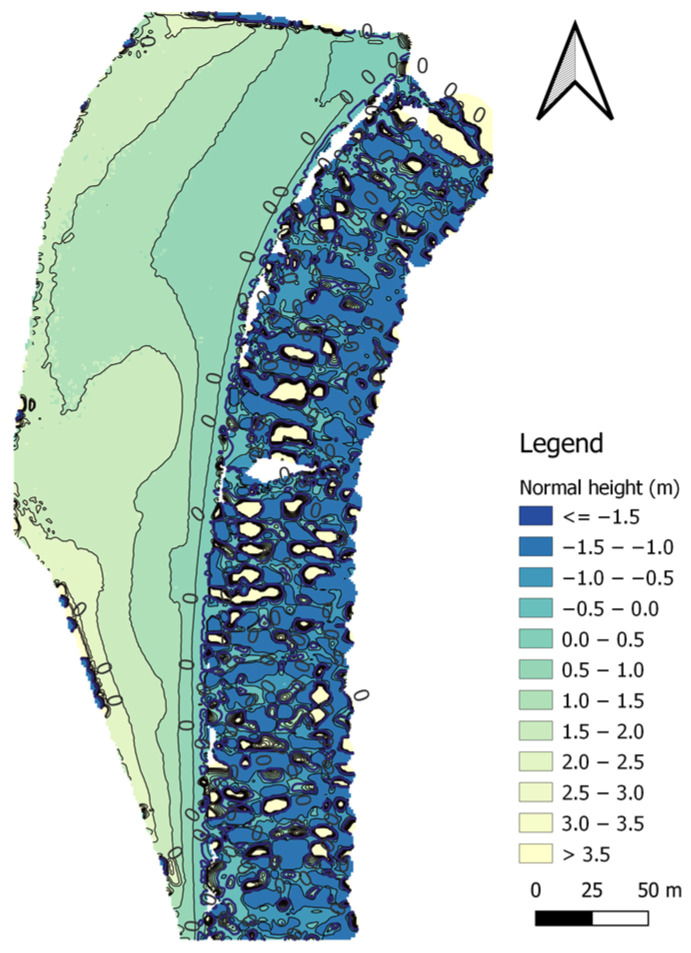
A topo-bathymetric chart of the waterbody adjacent to the public beach in Gdynia, obtained using the MSM method.

In the MSM method, it is very important to define the radius. This makes it possible to calculate the ellipse surface area and thus determine the range of data involved in the interpolation. The GRID model generated using the MSM method was obtained with the assumption that the two radii needed to define the ellipse are 46.2 m, and the number of points in each ellipse will be 13. The number of points needed to determine the weighting factors is 19. The topo-bathymetric model for the MSM method was created based on 34,166 measurement points and had only 31,810 nodes (out of 87,756 possible nodes), which were covered with heights. The vast majority of the nodes (37,914) were located on the land surface of the coastal zone. In total, 29,211 measurement points were used to model the topography of the land surface, which were compared with the corresponding points (with the same flat coordinates) from the interpolated DTM using the MSM method (Figure 8).

The RMS value of heights interpolated by using the MSM method was 1.668 m. The min. height value amounted to 0.001 m, while the max value was 3.856 m. The range amounted to 3.856 m, while the IRQ was 0.872 m.

The accuracy of the interpolated DTM in relation to the measurements was determined using the RMSE to be 0.100 m and the MAE to be 0.016 m. The RMSE and MAE values indicate a small difference between the interpolated and measurement values. The coefficient of determination was obtained at a level of 0.981, which means that the fit of the model to the measurement data is very good. The difference between the interpolated and measurement values for the R68 measure is 0.002 m, while for the R95 measure, it has a value of 0.030 m.

#### 3.1.4. NNI

**Figure 9:**
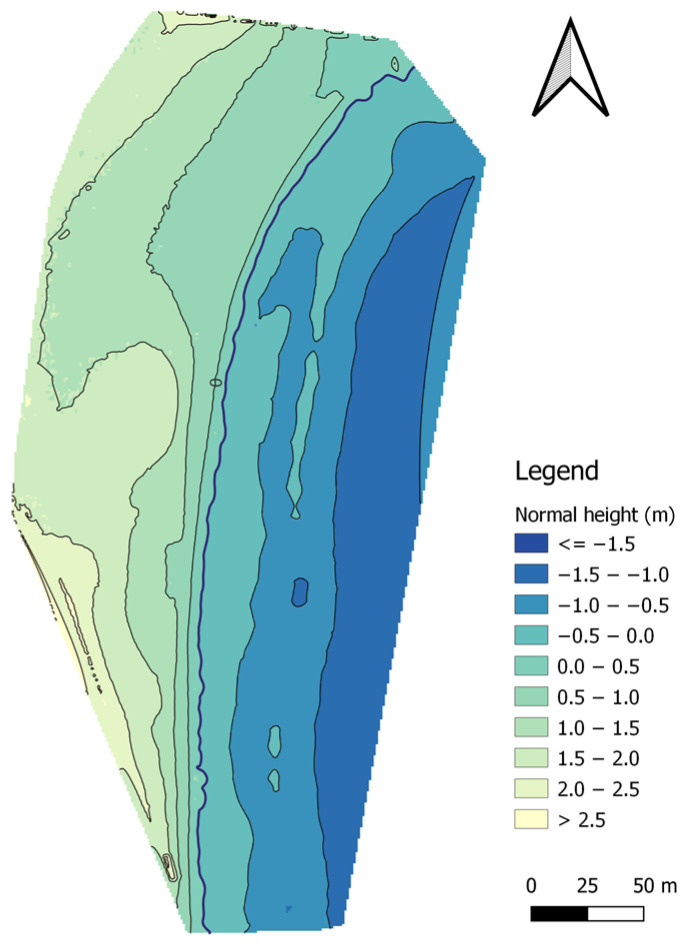
A topo-bathymetric chart of the waterbody adjacent to the public beach in Gdynia, obtained using the NNI method.

The NNI method is applied for input data that are evenly distributed; i.e., they are arranged in a regular grid. This means that the method can yield very good results for the topo-bathymetric model developed, e.g., based on the UAV data. This is due to the fact that the UAV data incorporated into the model are derived from the GRID model with a grid spacing of 1 m. The topo-bathymetric model for the NNI method was created based on 34,166 measurement points and had only 61,927 nodes (out of 87,756 possible nodes), which were covered with heights. Almost half of the nodes (30,685) were located on the land surface of the coastal zone. In total, 28,708 measurement points were used to model the topography of the land surface, which were compared with the corresponding points (with the same flat coordinates) from the interpolated DTM using the NNI method (Figure 9).

The RMS value of heights interpolated by using the NNI method was 1.140 m. The min. height value amounted to 0.000 m, while the max value was 3.418 m. The range amounted to 3.418 m, while the IRQ was 0.781 m.

The accuracy of the interpolated DTM in relation to the measurements was determined using the RMSE to be 0.032 m and the MAE to be 0.011 m. The RMSE and MAE values indicate a small difference between the interpolated and measurement values. The coefficient of determination was obtained at a level of 0.998, which means that the fit of the model to the measurement data is very good. The difference between the interpolated and measurement values for the R68 measure is 0.009 m, while for the R95 measure, it has a value of 0.032 m.

#### 3.1.5. Kriging (Logarithmic Model)

**Figure 10:**
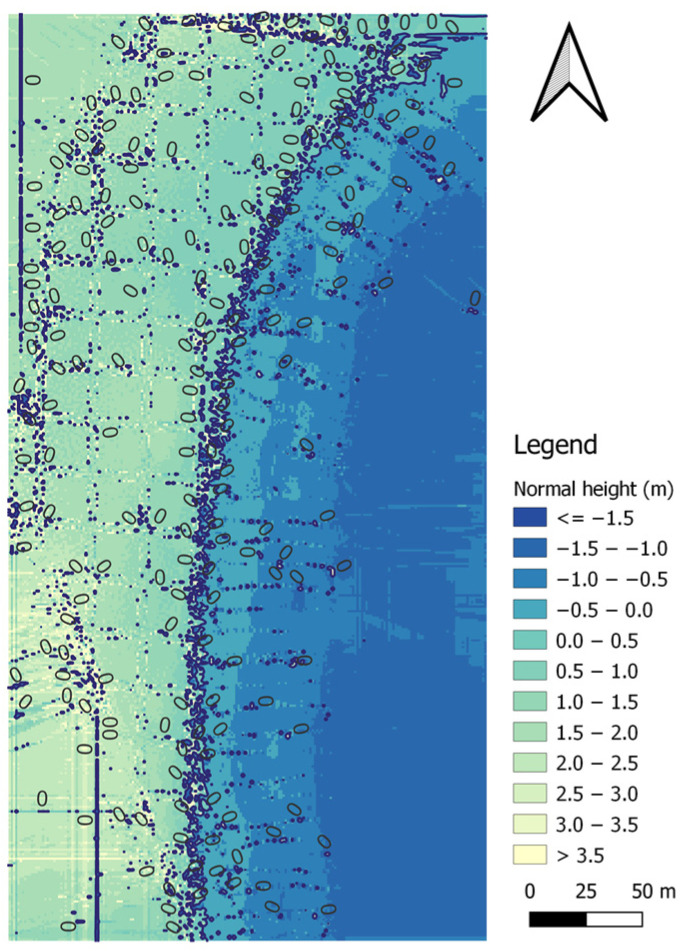
A topo-bathymetric chart of the waterbody adjacent to the public beach in Gdynia, obtained using the kriging (logarithmic model) method.

One of the most commonly applied surface interpolation methods is the kriging algorithm. A very important stage of this method is the selection of a semivariogram, i.e., a mathematical function to describe the empirical model most accurately. Therefore, we decided to select a logarithmic model at the beginning. Then, the radius determining the number of measurement points that have an effect on the point being interpolated was defined. The adopted values were the same as those for the previous methods. The topo-bathymetric model for the kriging (logarithmic model) method was created based on 34,166 measurement points and had 87,756 nodes, of which 41,878 nodes covered the land surface of the coastal zone. In total, 27,914 measurement points were used to model the topography of the land surface, which were compared with the corresponding points (with the same flat coordinates) from the interpolated DTM using the kriging (logarithmic model) method (Figure 10). Given the significant variation in the interpolated DTM, we decided to include only isobath 0 in the topo-bathymetric chart.

The RMS value of heights interpolated by using the kriging (logarithmic model) method was 1.746 m. The min. height value amounted to 0.000 m, while the max value was 3.857 m. The range amounted to 3.857 m, while the IRQ was 0.971 m.

The accuracy of the interpolated DTM in relation to the measurements was determined using the RMSE to be 0.263 m and the MAE to be 0.076 m. The RMSE and MAE values indicate a significant difference between the interpolated and measurement values. The coefficient of determination was obtained at a level of 0.176, which means that the fit of the model to the measurement data is poor. The difference between the interpolated and measurement values for the R68 measure is 0.262 m, while for the R95 measure, it has a value of 1.278 m.

#### 3.1.6. Kriging (Linear Model)

**Figure 11:**
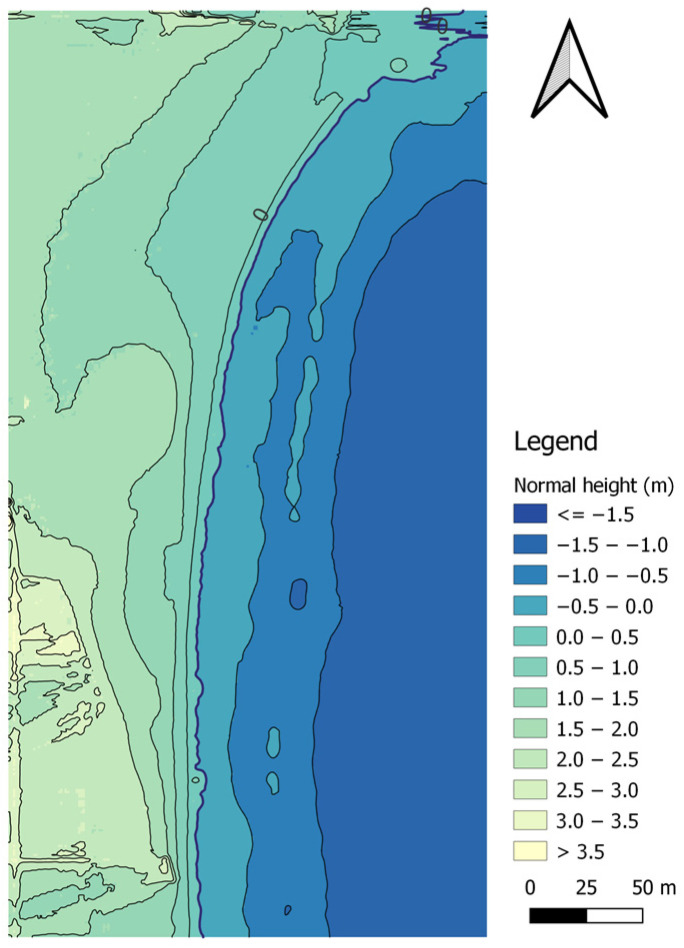
A topo-bathymetric chart of the waterbody adjacent to the public beach in Gdynia, obtained using the kriging (linear model) method.

The last method of land surface modelling was the kriging (linear model) algorithm. The topo-bathymetric model for the kriging (linear model) method was created based on 34,166 measurement points and had 87,756 nodes, of which 43,657 nodes covered the land surface of the coastal zone. In total, 29,030 measurement points were used to model the topography of the land surface, which were compared with the corresponding points (with the same flat coordinates) from the interpolated DTM using the kriging (linear model) method (Figure 11).

The RMS value of heights interpolated by using the kriging (linear model) method was 1.581 m. The min. height value amounted to 0.000 m, while the max value was 3.902 m. The range amounted to 3.902 m, while the IRQ was 0.798 m.

The accuracy of the interpolated DTM in relation to the measurements was determined using the RMSE to be 0.008 m and the MAE to be 0.000 m. The RMSE and MAE values indicate practically zero difference between the interpolated and measurement values. The coefficient of determination was obtained at a level of 0.996, which means that the fit of the model to the measurement data is very good. The difference between the interpolated and measurement values for the R68 measure is 0.008 m, while for the R95 measure, it has a value of 0.032 m. Based on the statistical analysis, it can be concluded that the selection of a semivariogram for the kriging method has a decisive influence on the accuracy of modelling land surfaces.

### 3.2. Modelling the Seabed Surface of the Coastal Zone

#### 3.2.1. IDP (*p* = 1)

On the seabed surface of the topo-bathymetric model generated by using the IDP (*p* = 1) method, there are 43,502 nodes. In total, 5136 measurement points were used to model the topography of the seabed surface, which were compared with the corresponding points (with the same flat coordinates) from the interpolated DTM using the IDP (*p* = 1) method (Figure 6).

The RMS value of heights interpolated by using the IDP (*p* = 1) method was 0.920 m. The min. height value amounted to –1.332 m, while the max value was 0.000 m. The range amounted to 1.332 m, while the IRQ was 0.661 m.

The accuracy of the interpolated DTM in relation to the measurements was determined using the RMSE to be 0.034 m and the MAE to be 0.025 m. The RMSE and MAE values indicate a small difference between the interpolated and measurement values. The coefficient of determination was obtained at a level of 0.995, which means that the fit of the model to the measurement data is very good. The difference between the interpolated and measurement values for the R68 measure is 0.028 m, while for the R95 measure, it has a value of 0.070 m.

#### 3.2.2. IDP (*p* = 2)

On the seabed surface of the topo-bathymetric model generated by using the IDP (*p* = 2) method, there are 43,292 nodes. In total, 5136 measurement points were used to model the topography of the seabed surface, which were compared with the corresponding points (with the same flat coordinates) from the interpolated DTM using the IDP (*p* = 2) method (Figure 7).

The RMS value of heights interpolated by using the IDP (*p* = 2) method was 0.923 m. The min. height value amounted to –1.346 m, while the max value was 0.000 m. The range amounted to 1.346 m, while the IRQ was 0.613 m.

The accuracy of the interpolated DTM in relation to the measurements was determined using the RMSE to be 0.019 m and the MAE to be 0.013 m. The RMSE and MAE values indicate a small difference between the interpolated and measurement values. The coefficient of determination was obtained at a level of 0.998, which means that the fit of the model to the measurement data is very good. The difference between the interpolated and measurement values for the R68 measure is 0.015 m, while for the R95 measure, it has a value of 0.038 m. Based on the statistical analysis, it can be concluded that, with the growth of the exponent in the IDP method, the accuracy of modelling the seabed surface of the coastal zone increases.

#### 3.2.3. MSM

For the UAV and USV data recorded on the waterbody adjacent to the public beach in Gdynia, the MSM model was applied with the assumption that the two radii needed to define the ellipse would each have a value of 46.2 m. This parameter enables the determination of the ellipse surface area, thus allowing the range of data involved in the interpolation to be determined. Furthermore, both the number of interpolating points located in the ellipse (13) and the number of points selected for the determination of weighting factors (19) were defined. The topo-bathymetric model for the MSM method was created based on 34,166 measurement points and had only 55,946 nodes (out of 87,756 possible nodes), which were covered with heights. Less than half of the nodes (18,032) were located on the seabed surface of the coastal zone. In total, 4895 measurement points were used to model the topography of the seabed surface, which were compared with the corresponding points (with the same flat coordinates) from the interpolated DTM using the MSM method (Figure 8).

The RMS value of heights interpolated by using the MSM method was 1.043 m. The min. height value amounted to –1.370 m, while the max value was 0.000 m. The range amounted to 1.370 m, while the IRQ was 0.819 m.

The accuracy of the interpolated DTM in relation to the measurements was determined using the RMSE to be 0.123 m and the MAE to be 0.061 m. The RMSE and MAE values indicate a visible difference between the interpolated and measurement values. The coefficient of determination was obtained at a level of 0.931, which means that the fit of the model to the measurement data is good. The difference between the interpolated and measurement values for the R68 measure is 0.047 m, while for the R95 measure, it has a value of 0.262 m.

#### 3.2.4. NNI

The NNI method is one that does not always cover the entire area with the modelled data. This is due to the fact that the point under interpolation is interpolated using only those points for which the interpolated point can be attached to the side of a triangle from the triangle network. Nevertheless, this method allows very accurate surface models to be generated. The topo-bathymetric model for the NNI method was created based on 34,166 measurement points and had only 61,927 nodes (out of 87,756 possible nodes), which were covered with heights. Around half of the nodes (31,242) were located on the seabed surface of the coastal zone. In total, 5036 measurement points were used to model the topography of the seabed surface, which were compared with the corresponding points (with the same flat coordinates) from the interpolated DTM using the NNI method (Figure 9).

The RMS value of heights interpolated by using the NNI method was 0.799 m. The min. height value amounted to –1.365 m, while the max value was 0.000 m. The range amounted to 1.365 m, while the IRQ was 0.591 m.

The accuracy of the interpolated DTM in relation to the measurements was determined using the RMSE to be 0.020 m and the MAE to be 0.002 m. The RMSE and MAE values indicate a small difference between the interpolated and measurement values. The coefficient of determination was obtained at a level of 0.998, which means that the fit of the model to the measurement data is very good. The difference between the interpolated and measurement values for the R68 measure is 0.014 m, while for the R95 measure, it has a value of 0.039 m.

#### 3.2.5. Kriging (Logarithmic Model)

In the seabed surface of the topo-bathymetric model generated by the kriging (logarithmic model) method, there are 45,878 nodes. In total, 6252 measurement points were used to model the topography of the seabed surface, which were compared with the corresponding points (with the same flat coordinates) from the interpolated DTM using the kriging (logarithmic model) method (Figure 10).

The RMS value of heights interpolated by using the kriging (logarithmic model) method was 0.931 m. The min. height value amounted to –1.370 m, while the max value was 0.000 m. The range amounted to 1.370 m, while the IRQ was 0.612 m.

The accuracy of the interpolated DTM in relation to the measurements was determined using the RMSE to be 0.808 m and the MAE to be 0.447 m. The RMSE and MAE values indicate a significant difference between the interpolated and measurement values. The coefficient of determination was obtained at a level of 0.410, which means that the fit of the model to the measurement data is very bad. The difference between the interpolated and measurement values for the R68 measure is 0.338 m, while for the R95 measure, it has a value of 2.073 m.

#### 3.2.6. Kriging (Linear Model)

In the seabed surface of the topo-bathymetric model generated by the kriging (linear model) method, there are 44,099 nodes. In total, 5136 measurement points were used to model the topography of the seabed surface, which were compared with the corresponding points (with the same flat coordinates) from the interpolated DTM using the kriging (linear model) method (Figure 11).

The RMS value of heights interpolated by using the kriging (linear model) method was 0.949 m. The min. height value amounted to –1.445 m, while the max value was 0.000 m. The range amounted to 1.445 m, while the IRQ was 0.693 m.

The accuracy of the interpolated DTM in relation to the measurements was determined using the RMSE to be 0.018 m and the MAE to be 0.000 m. The RMSE and MAE values indicate a slight difference between the interpolated and measurement values. The coefficient of determination was obtained at a level of 0.998, which means that the fit of the model to the measurement data is very good. The difference between the interpolated and measurement values for the R68 measure is 0.012 m, while for the R95 measure, it has a value of 0.034 m. Based on the statistical analysis, it can be concluded that the selection of a semivariogram for the kriging method has a decisive influence on the accuracy of modelling the seabed surface.

## 4. Discussion

The aim of this article is to present land and seabed surface modelling in the coastal zone using UAV/USV-based data integration. Bathymetric and photogrammetric measurements were carried out on the waterbody adjacent to the public beach in Gdynia (Poland) in 2022 using the DJI Phantom 4 RTK UAV and the AutoDron USV. As a result of geospatial data integration, topo-bathymetric models in the coastal zone were developed using the following terrain-modelling methods: IDP, kriging, MSM and NNI.

Based on Table 2, it can be concluded that the highest max height for the land part was obtained using the kriging (linear model) method, and the lowest max height was noted for the IDP (*p* = 1) method. In turn, the RMS measure yielded the highest value for the kriging (logarithmic model) method and the lowest value for the NNI method.

As for the GRID models only, including the seabed part (Table 3), the highest min. value was noted for the IDP (*p* = 1) method, and the lowest min. value was for the kriging (linear model) method. In turn, the RMS measure yielded the highest value for the MSM method and the lowest value for the NNI method.

However, the crucial stage of the ongoing work is to assess the accuracies of selected topo-bathymetric models obtained using different interpolation methods, taking into account the division into land and seabed parts. It should be remembered that the IDW method was not considered, as the IDW method-generated model of the waterbody adjacent to the public beach in Gdynia only comprised heights equal to or higher than 0 m. According to the statistical analysis conducted for the land part, the best results were obtained by using the kriging (linear model) method (Table 4). This indicates very low values obtained for the RMSE (0.008 m) and MAE (0.000 m) measures. However, the highest coefficient of determination value (0.998) was noted for the NNI method. Moreover, for the MSM model, the R68 measure had a value of 0.002 m, and the R95 measure was 0.030 m.

Subsequently, we decided to analyse the accuracies of the seabed surface modelling of the coastal zone (Table 5). The lowest RMSE value (0.018 m) was noted for the kriging (linear model) method, and the second lowest value (0.019 m) was obtained for the IDP (*p* = 2) method. Moreover, the lowest MAE value (0.000 m) was noted for the kriging (linear model) method. The best fit of the model to the data according to the coefficient of determination (0.998) was obtained for the IDP (*p* = 2), NNI and kriging (linear model) methods. The lowest values for the R68 (0.012 m) and R95 (0.034 m) measures were noted for the kriging (linear model) method.

It can be noted that the best accuracies for both the land and seabed parts were obtained for the GRID model generated by the kriging (linear model) method. It is noteworthy that the NNI method obtained a very high value for the coefficient of determination (0.998) of both the land and seabed parts of the coastal zone.

The presented article is a continuation of the research in [56], the purpose of which was to assess the accuracy of topo-bathymetric surface models based on geospatial data recorded by UAVs and USVs. According to the mentioned studies, having compared the accuracy measures of the charts and models obtained, it was concluded that the kriging (linear model) method is best. The accuracy of the interpolated DTM created by the kriging (linear model) method in relation to the measurements was determined using the RMSE to be 0.030 m and the MAE to be 0.011 m. The coefficient of determination was obtained at a level of 0.999. The difference between the interpolated value and the measurement value for the R68 measure is 0.009 m, while for the R95 measure, it has a value of 0.033 m. The results of the conducted experiments show that the most accurate method of surface modelling (land; water; land and water) is the kriging (linear model) method.

## 5. Conclusions

Creating a topo-bathymetric model of the coastal zone requires first obtaining data using various hydroacoustic and optoelectronic devices, thus providing data with different land cover densities. For the area covering the waterbody adjacent to the public beach in Gdynia, including the beach, there were significantly more data obtained using a UAV (28,990 points) than data measured using an SBES (5136 points). Therefore, it can be assumed that the topo-bathymetric model of the land surface of the coastal zone will be more accurate than the seabed surface.

This publication is an analysis of selected accuracy measures obtained for the topo-bathymetric model separately for land and seabed surfaces. Based on the analysis carried out, it can be concluded that the most accurate modelling method for both the land and seabed surfaces is kriging (linear method). The RMSE for the land surface modelled using the kriging method was 0.008 m. For the seabed surface, it was 0.018 m. Moreover, the R68 measure has a value of 0.008 m for the land surface and 0.012 m for the seabed surface. The R95 measure amounts to 0.032 m for the land surface and 0.034 m for the seabed surface.

The presented example was used for an area where measurements were carried out using a UAV and a USV equipped with an SBES. In the future, the same analysis should be performed but based on bathymetric data measured by an MBES. Then, it will be possible to clearly determine whether data coverage has an impact on the accuracy of the modelled surfaces of the land and seabed in the coastal zone.

## Figures and Tables

**Table 1:** Georeference point coordinates in the PL-UTM/PL-EVRF2007-NH systems and their differences in relation to point coordinates in the UAV point cloud.

No.	Easting (m)	Northing (m)	*H_PL-EVRF2007-NH_* (m)	*dE* ^1^ (m)	*dN* ^2^ (m)	*dHn* ^3^ (m)
1	4,341,518.944	6,043,718.866	0.607	0.007	−0.009	−0.009
2	4,341,482.962	6,043,652.071	0.789	0.009	0.008	0.004
3	4,341,473.488	6,043,610.972	0.875	−0.017	−0.003	−0.015
4	4,341,467.661	6,043,567.572	0.908	0.002	0.001	0.007
5	4,341,462.424	6,043,509.593	0.990	−0.006	0.000	−0.009
6	4,341,461.894	6,04,3452.436	0.932	0.022	0.010	0.024
			*σ*	0.012	0.006	0.013

Differences between the easting ^1^, northing ^2^ and normal-height ^3^ coordinates of the georeference points in the PL-UTM/PL-EVRF2007-NH systems and the points in the UAV point cloud recorded in the WGS-84/UTM systems.

**Table 2:** A summary of information in the GRID-generated models for the land part.

Statistical Measure (m)	IDP (*p* = 1)	IDP (*p* = 2)	MSM	NNI	Kriging (Logarithmic Model)	Kriging (Linear Model)
*h_max_* ^1^	2.690	3.102	3.856	3.418	3.857	3.902
*h_min_* ^2^	0.000	0.000	0.001	0.000	0.000	0.000
RMS	1.527	1.522	1.668	1.140	1.746	1.581
R	2.690	3.102	3.856	3.418	3.857	3.902
IRQ	0.828	0.851	0.872	0.781	0.971	0.798

The max ^1^ and min. ^2^ values of the height coordinates in the GRID model.

**Table 3:** A summary of information in the GRID-generated models for the seabed part.

Statistical Measure (m)	IDP (*p* = 1)	IDP (*p* = 2)	MSM	NNI	Kriging (Logarithmic Model)	Kriging (Linear Model)
*h_max_* ^1^	0.000	0.000	0.000	0.000	0.000	0.000
*h_min_* ^2^	–1.332	–1.346	–1.370	–1.365	–1.370	–1.445
RMS	0.920	0.923	1.403	0.799	0.931	0.949
R	1.332	1.346	1.370	1.365	1.370	1.445
IRQ	0.661	0.613	0.819	0.591	0.612	0.693

The max ^1^ and min. ^2^ values of the depth coordinates in the GRID model.

**Table 4:** The accuracies of the land surface modelling of the coastal zone.

Statistical Measure (m)	IDP (*p* = 1)	IDP (*p* = 2)	MSM	NNI	Kriging (Logarithmic Model)	Kriging (Linear Model)
RMSE (m)	0.061	0.042	0.100	0.032	0.263	0.008
MAE (m)	0.023	0.016	0.016	0.011	0.076	0.000
R^2^ (–)	0.992	0.996	0.981	0.998	0.176	0.996
R68 (m)	0.019	0.013	0.002	0.009	0.262	0.008
R95 (m)	0.072	0.048	0.030	0.032	1.278	0.032

**Table 5:** The accuracies of the seabed surface modelling of the coastal zone.

Statistical Measure (m)	IDP (*p* = 1)	IDP (*p* = 2)	MSM	NNI	Kriging (Logarithmic Model)	Kriging (Linear Model)
RMSE (m)	0.034	0.019	0.123	0.020	0.808	0.018
MAE (m)	0.025	0.013	0.061	0.002	0.447	0.000
R^2^ (–)	0.995	0.998	0.931	0.998	0.410	0.998
R68 (m)	0.028	0.015	0.047	0.014	0.338	0.012
R95 (m)	0.070	0.038	0.262	0.039	2.073	0.034
